# Advanced Oxidation Protein Products Induce G1/G0-Phase Arrest in Ovarian Granulosa Cells via the ROS-JNK/p38 MAPK-p21 Pathway in Premature Ovarian Insufficiency

**DOI:** 10.1155/2021/6634718

**Published:** 2021-07-27

**Authors:** Xing-Yu Zhou, Jun Zhang, Ying Li, Ying-Xue Chen, Xiao-Min Wu, Xin Li, Xiao-Fei Zhang, Lin-Zi Ma, Yi-Zhen Yang, Ke-Ming Zheng, Yu-Dong Liu, Zhe Wang, Shi-Ling Chen

**Affiliations:** Center for Reproductive Medicine, Department of Obstetrics and Gynecology, Nanfang Hospital, Southern Medical University, No 1838 Guangzhou Northern Road, Guangzhou 510515, China

## Abstract

The mechanism underlying the role of oxidative stress and advanced oxidation protein products (AOPPs) in the aetiology of premature ovarian insufficiency (POI) is poorly understood. Here, we investigated the plasma AOPP level in POI patients and the effects of AOPPs on granulosa cells both in vitro and in vivo. KGN cells were treated with different AOPP doses, and cell cycle distribution, intracellular reactive oxygen species (ROS), and protein expression levels were measured. Sprague–Dawley (SD) rats were treated daily with PBS, rat serum albumin, AOPP, or AOPP+ N-acetylcysteine (NAC) for 12 weeks to explore the effect of AOPPs on ovarian function. Plasma AOPP concentrations were significantly higher in both POI and biochemical POI patients than in controls and negatively correlated with anti-Müllerian hormone and the antral follicle count. KGN cells treated with AOPP exhibited G1/G0-phase arrest. AOPP induced G1/G0-phase arrest in KGN cells by activating the ROS-c-Jun N-terminal kinase (JNK)/p38 mitogen-activated protein kinase (MAPK)-p21 pathway. Pretreatment with NAC, SP600125, SB203580, and si-p21 blocked AOPP-induced G1/G0-phase arrest. In SD rats, AOPP treatment increased the proportion of atretic follicles, and NAC attenuated the adverse effects of AOPPs in the ovary. In conclusion, we provide mechanistic evidence that AOPPs may induce cell cycle arrest in granulosa cells via the ROS-JNK/p38 MAPK-p21 pathway and thus may be a novel biomarker of POI.

## 1. Introduction

Premature ovarian insufficiency (POI) is a disease in which the ovarian reserve is exhausted prematurely before the age of 40 and is characterized by sterility, amenorrhea or oligomenorrhea, hypoestrogenism, and elevated serum follicle-stimulating hormone (FSH) concentrations (>25 mIU/ml). Women who suffer from POI for a long period of time are at increased risk for cardiovascular diseases, neurological function impairment, psychological dysfunction, and osteoporosis [1]. However, there are currently no effective disease-modifying therapies for POI, and its pathogenesis remains poorly understood.

Reactive oxygen species (ROS) are the most important hyperactive molecules during the process of oxidative stress. Although low levels of ROS are essential for biological functions in cells and tissues, such as ovulation and luteolysis in the ovary [2], elevated production of ROS beyond the antioxidant capacity is detrimental to cell function and participates in various human diseases, including diabetes [3], cardiovascular disorders [4], and neurological disorders [5]. Recent studies have reported that POI manifests with elevated serum levels of ROS and oxidative stress markers [6, 7], while excessive oxidative stress can lead to impairment of ovarian function [8]. However, the mechanism underlying the role of oxidative stress in the aetiology of POI has received limited attention.

Advanced oxidation protein products (AOPPs) are novel protein markers of oxidation-mediated protein damage and have been reported to accumulate in many chronic inflammatory diseases, such as chronic kidney disease [9], osteoporosis [10], and inflammatory bowel disease [11]. AOPPs are altered in the peritoneal fluid and follicular fluid of patients with endometriosis, and significant negative correlations between AOPP concentrations in follicular fluid and blastocyst rates have been observed [12–14]. In addition to being products of chronic oxidative stress, AOPPs can trigger oxidative stress and induce a variety of cell pathological changes, including apoptosis [15], autophagy [16], and pyroptosis [17]. An increasing number of studies have revealed that supraphysiological AOPP levels contribute to cell cycle arrest in intestinal epithelial cells [18] and hepatocytes [19] by elevating ROS levels and thus activating ROS-mediated downstream pathways. However, whether AOPP levels are increased in women with POI and whether the pathophysiological concentration of AOPPs adversely affects granulosa cells and ovarian function has not been reported.

The present study first provides in vivo and in vitro evidence that AOPP accumulation inhibits cell proliferation by activating the ROS-c-Jun N-terminal kinase (JNK)/p38 mitogen-activated protein kinase (MAPK)-p21 signaling pathway in granulosa cells, thus, suggesting that enhanced AOPP accumulation may exert pathobiological effects in granulosa cells and participate in the pathogenesis of POI.

## 2. Materials and Methods

### 2.1. Patient Samples and Inclusion Criteria

The study was conducted in accordance with the Declaration of Helsinki, and the protocol was approved by the Ethics Committee of Nanfang Hospital, Southern Medical University. Written informed consent was obtained from all patients. Venous blood for this study was collected from 61 patients with POI, 21 patients with biochemical POI (bPOI), and 30 age- and body mass index- (BMI-) matched women with normal ovarian reserve at the Center for Reproductive Medicine, Department of Gynecology and Obstetrics at Nanfang Hospital, People's Republic of China, from August 2018 to July 2020. POI diagnosis was based on the European Society of Human Reproduction and Embryology (ESHRE) guidelines [1]. The inclusion criteria for bPOI patients were described in our previous study, including (1) <40 years of age, (2) basal FSH (on days 2-4 of menstrual cycle) ≥10 mIU/ml, (3) bilateral ovarian antral follicle count (AFC) ≤ 7, (4) anti-Müllerian hormone (AMH) ≤ 1.0 ng/ml, and (5) having spontaneous menstruation [20]. The controls were women who had undergone in vitro fertilization (IVF)/intracytoplasmic sperm injection (ICSI) treatment with male factor infertility or tubal obstruction, were under 40 years of age, and had regular menstrual cycles (25–35 days) with normal ovarian morphology and ovarian reserve (FSH < 10 IU/mL on menstrual cycle days 2–4, bilateral AFC > 8, and AMH concentrations > 1.1 ng/mL). Women with abnormal karyotypes; a history of other endocrine diseases such as polycystic ovary syndrome (PCOS), hyperprolactinemia, and hyperthyroidism; a history of radiotherapy, chemotherapy, or ovarian operation; or other disorders in which AOPP are reported to be increased were excluded from all groups. The blood samples were centrifuged at 2000 × g for 10 min, and the supernatant was centrifuged again at 13000 × g for 10 min to remove platelets. Then, the serum was collected and kept frozen at −80°C until assessment.

### 2.2. AOPP Determination

Plasma AOPP concentrations were assessed as chloramine-T equivalents as described previously [18]. Briefly, 200 *μ*L of each plasma sample (diluted 5 times in PBS) was placed into a 96-well plate, and then 10 *μ*L of 1.16 M potassium iodide (Sigma, St. Louis, MO, USA) and 20 *μ*L of acetic acid (Sigma, St. Louis, MO, USA) were added. Two hundred microlitres of chloramine-T (Sigma) at different concentrations (0, 20, 40, 80, and 100 *μ*M) were used for calibration. The absorbance at 340 nm was read immediately by a Spectra Max M5 spectrophotometer (Molecular Devices, San Francisco, CA, USA). The AOPP concentrations are expressed as *μ*mol/L of chloramine-T equivalents.

### 2.3. AOPP Preparation

Bovine serum albumin (BSA) or rat serum albumin (RSA) (20 mg/mL; Sigma) was incubated with 40 mM HOCl (Sigma) for 30 min at room temperature, and the mixture was then dialyzed in PBS for 24 h at 4°C to remove free HOCl [10]. Unmodified BSA or RSA was dissolved in PBS alone as a control.

### 2.4. Cell Culture and Cell Transfection

KGN cells were purchased from the RIKEN BioResource Center (Tsukuba, Japan) and maintained at 37°C with a 5% CO2 atmosphere in Dulbecco's modified Eagle's medium (DMEM)/F-12 nutrient mixture supplemented with 10% foetal bovine serum (Gibco, Life Technologies, Carlsbad, CA, USA). Oligonucleotide siRNA duplexes were synthesized by RiboBio (Guangzhou, China). KGN cells were transfected with siRNAs using Lipofectamine RNAiMax (Life Technologies, Carlsbad, CA, USA).

### 2.5. Culture of Human Luteinized Granulosa Cells (hLGC)

hLGC were isolated from the follicular fluid as previously described [20], then 1-2 ml of 2% hyaluronidase-containing in DMEM/F12 digestion solution was used to digest at 37°C for 10 min. The digested cells were collected by centrifugation at 1000 × rpm for 5 min after digestion termination. Separated cells were plated in a 96-well plate to detect cell activity.

### 2.6. Cell Counting Kit-8 (CCK-8) Assay

A CCK-8 assay (Dojindo Laboratories, Kumamoto, Japan) was applied to detect cell proliferation every 12 h. KGN cells were plated in 96-well plates (3000 cells/well) with AOPP at different concentrations of 50, 100, 200, and 300 *μ*g/mL for 12 h, 24 h, 36 h, and 48 h. Two hours before absorbance measurement, 10 *μ*L of CCK-8 solution was added to each well, and then the optical density was measured at 450 nm using spectrophotometry (Spectra Max M5). Each experiment was repeated three times with five biological replicates each time.

### 2.7. Ethynyl-2-Deoxyuridine (EdU) Assay

An EdU assay (RiboBio) was performed following the manufacturer's instructions. In brief, cells were cultured in 96-well plates (3000 cells/well) for 24 h, and then AOPP at different concentrations of 50, 100, 200, and 300 *μ*g/mL was added to the cells. Approximately 24 h after AOPP stimulation, 50 *μ*M EdU labelling medium was added to each well, and the cells were incubated for 2 h. The culture medium was removed, and then the cells were washed with PBS. Four percent polyformaldehyde was used to fix the cells for 30 min and was then neutralized with 2 mg/mL glycine for 5 min. Subsequently, the cells were permeabilized with 0.5% Triton X and treated with 1× Apollo solution and Hoechst 33342 for 30 min at room temperature in the dark. Images were captured using an Olympus LX71 fluorescence inverted microscope (Olympus, Tokyo, Japan), and the percentage of EdU-positive cells was calculated from five random fields in three wells.

### 2.8. Flow Cytometry Analysis

To assess cell cycle distribution, KGN cells were trypsinized, resuspended in 70% ethanol, and incubated overnight at 4°C. The fixed cells were centrifuged, washed in ice-cold PBS, and then incubated in RNase A at 37°C for 30 min and in 400 *μ*L of propidium iodide (BestBio, Shanghai, China) at 4°C for 30 min. A FACScan flow cytometer (BD Biosciences, San Jose, CA, USA) and ModFit 3.0 software (Verity Software House, Topsham, ME, USA) were used for analysis.

### 2.9. Determination of Intracellular ROS Generation

KGN cells were incubated with 10 *μ*M 2′,7′-dichlorofluorescein diacetate (DCFH-DA) (Sigma) for 30 min at 37°C to detect intracellular ROS. Fluorescence intensity (Ex/Em = 488/525) was measured on a fluorescence microplate reader (Spectra Max M5).

### 2.10. RNA Isolation and Quantitative Real-time Polymerase Chain Reaction (qRT-PCR)

Total RNA was isolated using TRIzol reagent (Takara, Dalian, China) in accordance with the manufacturer's instructions. The first-strand cDNA was reverse transcribed using a PrimeScript RT Reagent Kit with gDNA Eraser (Takara), and qRT-PCR was performed using a SYBR Green PCR kit (Takara) on a LightCycler 480 (Roche Molecular Biochemicals, Basel, Switzerland). The *β*-tubulin gene was used as the internal control for the quantification of target genes. Primer sequences are listed as follows: *β*-tubulin forward 5′-GGCCAAGGGTCACTACACG-3′, reverse 5′-GCAGTCGCAGTTTTCACACTC-3′; cyclin E1 forward 5′-GAACTGTGTCAAGTGGATGGT-3′, reverse 5′-CCGCTGCTCTGCTTCTTAC-3′; CDK2 forward 5′-CCAGGAGTTACTTCTATGCCTGA-3′, reverse 5′-TTCATCCAGGGGAGGTACAAC-3′; p21 forward 5′-TGTCCGTCAGAACCCATGC-3′, reverse 5′-AAAGTCGAAGTTCCATCGCTC-3′.

### 2.11. Western Blot Analysis

Cultured cells were lysed in ice-cold radio-immunoprecipitation assay (RIPA) buffer with 1× phenylmethanesulfonyl fluoride (PMSF), 1× protease inhibitor cocktail, and 1× phosphatase inhibitor (Beyotime, Shanghai, China). The lysates were cleared by centrifugation at 13000 rpm and 4°C for 15 min, and a bicinchoninic acid (BCA) Protein Assay Kit (Beyotime) was used to determine the protein concentration of the supernatant. The proteins in the lysates were then separated by SDS-polyacrylamide gel electrophoresis (PAGE) with 8%–12% acrylamide gels and transferred to polyvinylidene fluoride (PVDF) membranes (Bio-Rad Laboratories, Hercules, CA, USA). The membranes were blocked using 5% BSA for 1 h at room temperature and then incubated with primary antibodies overnight at 4°C. After being washed 3 times with Tris-buffered saline (TBS) with 0.1% Tween 20 (TBST) for 10 min, the membranes were incubated with horseradish peroxidase- (HRP-) conjugated secondary antibodies (Cell Signaling Technology (CST), Beverly, MA, USA) for 1 h at room temperature and then washed again 3 times with TBST. Immunoreactive proteins were visualized with Clarity Western Enhanced Chemiluminescence (ECL) Substrate (Bio-Rad Laboratories). ImageJ software was used to analyse and normalize the band intensities. The following primary antibodies were used: anti-mouse cyclin E1 (4129, CST), anti-rabbit CDK2 (2546, CST), anti-rabbit p21 (2947, CST), anti-rabbit phospho-JNK (4668, CST), anti-rabbit JNK (9252, CST), anti-rabbit phospho-p38 MAPK (4511, CST), anti-rabbit p38 MAPK (8690, CST), anti-rabbit phospho-ERK1/2 (4370, CST), anti-rabbit ERK1/2 (4695, CST), anti-rabbit *β*-tubulin (2128, CST), anti-mouse p22^phox^ (sc-271968, Santa Cruz Biotechnology, Dallas, TX, USA), anti-mouse p47^phox^ (sc-17844, Santa Cruz Biotechnology), anti-mouse gp91^phox^ (sc-130543, Santa Cruz Biotechnology), and anti-rabbit NOX4 (ab109225, Abcam, Cambridge, UK) antibodies.

### 2.12. Immunoprecipitation

An immunoprecipitation kit (Proteintech, Wuhan, China) was utilized to evaluate the interactions of p22^phox^ with p47^phox^ and of p22^phox^ with NOX4 in KGN cells according to the manufacturer's instructions. In brief, cell lysates were preincubated with anti-mouse p22^phox^ (sc-271968, Santa Cruz Biotechnology) or anti-mouse p47^phox^ antibodies (sc-17844, Santa Cruz Biotechnology) at 4°C overnight and incubated with Protein A Sepharose beads at 4°C for 4 h. Protein complexes were obtained after washed and eluted 5 times, and then detected by western blotting using anti-mouse p22^phox^ (sc-271968, Santa Cruz Biotechnology), anti-mouse p47^phox^ (sc-17844, Santa Cruz Biotechnology), and anti-rabbit NOX4 (ab109225, Abcam) antibodies.

### 2.13. Immunofluorescence Staining

KGN cells were cultured on confocal dishes, fixed with 4% paraformaldehyde for 30 min, and permeabilized with 0.5% Triton X-100 for 15 min. Then, the cells were blocked using 5% BSA for 1 h and incubated with anti-mouse p47^phox^ antibodies (Santa Cruz Biotechnology) overnight at 4°C. After that, the cells were washed with PBS three times and stained with Alexa Fluor 488-labelled IgG (Beyotime) for 1 h and DAPI for 5 min. The cells were imaged on a Zeiss LSM 880 with Airyscan confocal microscope (Carl Zeiss Microscopy GmbH, Jena, Germany).

### 2.14. Animal Experiments

All animal studies were approved by the Laboratory of Animal Care and Use Committee of Southern Medical University. Thirty-six female Sprague–Dawley (SD) rats (7 weeks, weighing 200–230 g) were housed in the Southern Medical University Animal Experiment Center (Guangzhou, China) under standard environmental conditions with 12 h : 12 h light/dark cycles and free access to water and food. All rats were randomly divided into four treatment groups (*n* = 9 per group): the PBS group, with daily intraperitoneal injection of PBS (pH = 7.4); the RSA group, with daily intraperitoneal injection of unmodified RSA (50 mg/kg); the AOPP group, with daily intraperitoneal injection of AOPP (50 mg/kg); and the AOPP+N-acetylcysteine (NAC) group, with daily intraperitoneal injection of both AOPP (50 mg/kg) and NAC (200 mg/kg). The rats were anaesthetized with isoflurane and sacrificed at the end of week 12. Their ovaries were rapidly dissected and washed with ice-cold saline. Then, the left ovaries were stored at −80°C, and the right ovaries were fixed in 4% formaldehyde. Their blood was collected via cardiocentesis.

### 2.15. Morphological and Immunohistochemical Analyses

Ovaries were fixed in 4% paraformaldehyde for 24 h, dehydrated in ethanol, and embedded in paraffin wax. Sections 4 *μ*m thick were deparaffinized with xylene and stained with haematoxylin and eosin (H&E) following standard protocols for follicle assessment. The slides were examined and photographed using a Leica CS2 slide scanner system (Leica, Wetzlar, Germany). A follicle containing an oocyte with a distinct nucleus was counted and classified as a primordial, primary, secondary, antral, or atretic follicle according to previously described criteria [21]. The number of follicles was calculated from the total counts of three representative ovarian midsections of each rat. The proportion of each follicle stage in the different treatment group was calculated.

### 2.16. Immunohistochemical Analysis

Paraffin-embedded ovary tissue was sectioned at 4 *μ*m thickness, deparaffinized in xylene, and rehydrated in a graded series of alcohols. After antigen retrieval, 3% hydrogen peroxide was used to block endogenous peroxidase. The sections were incubated in serum for blocking and subsequently incubated with primary antibodies against proliferating cell nuclear antigen (PCNA, 13110, CST), p-JNK (4668, CST), p-p38 MAPK (4511, CST), p21 (ab80633, Abcam), p22^phox^ (sc-271968, Santa Cruz Biotechnology), and p47^phox^ (sc-17844, Santa Cruz Biotechnology) overnight at 4°C before being incubated with a secondary antibody and treated with a diaminobenzidine (DAB) staining kit (Zhongshanjinqiao, Beijing, China). The slides were examined with an Olympus BX63 microscope.

### 2.17. Serum Hormone Measurement with Enzyme-Linked Immunosorbent Assays (ELISA)

Rat blood samples were kept at 4°C overnight and then centrifuged at 3000 × g for 10 min, and the serum was kept frozen at −80°C until assessment. The serum levels of the hormones AMH, FSH, and estradiol were measured with ELISA kits (Enzyme-linked Biotechnology, Shanghai, China).

### 2.18. Statistical Analyses

All experimental data are presented as the means ± standard deviations and were analysed with the SPSS 20.0 (International Business Machines Corporation (IBM), Armonk, NY, USA) and GraphPad Prism 6 (GraphPad Software, San Diego, CA, USA) software programs. Differences between two groups were determined via independent-sample Student's *t*-tests for quantitative data with a Gaussian distribution or Mann–Whitney *U* tests for data with a non-Gaussian distribution. For comparisons among more than two groups, a one-way analysis of variance (ANOVA) was conducted with the Bonferroni post hoc test. Pearson correlation and linear regression analyses were applied to assess the correlations between plasma AOPP levels and ovarian reserve indicators. A value of <0.05 was considered to indicate statistical significance.

## 3. Results

### 3.1. Plasma AOPPs Accumulate in Both POI and bPOI

**Figure 1:**
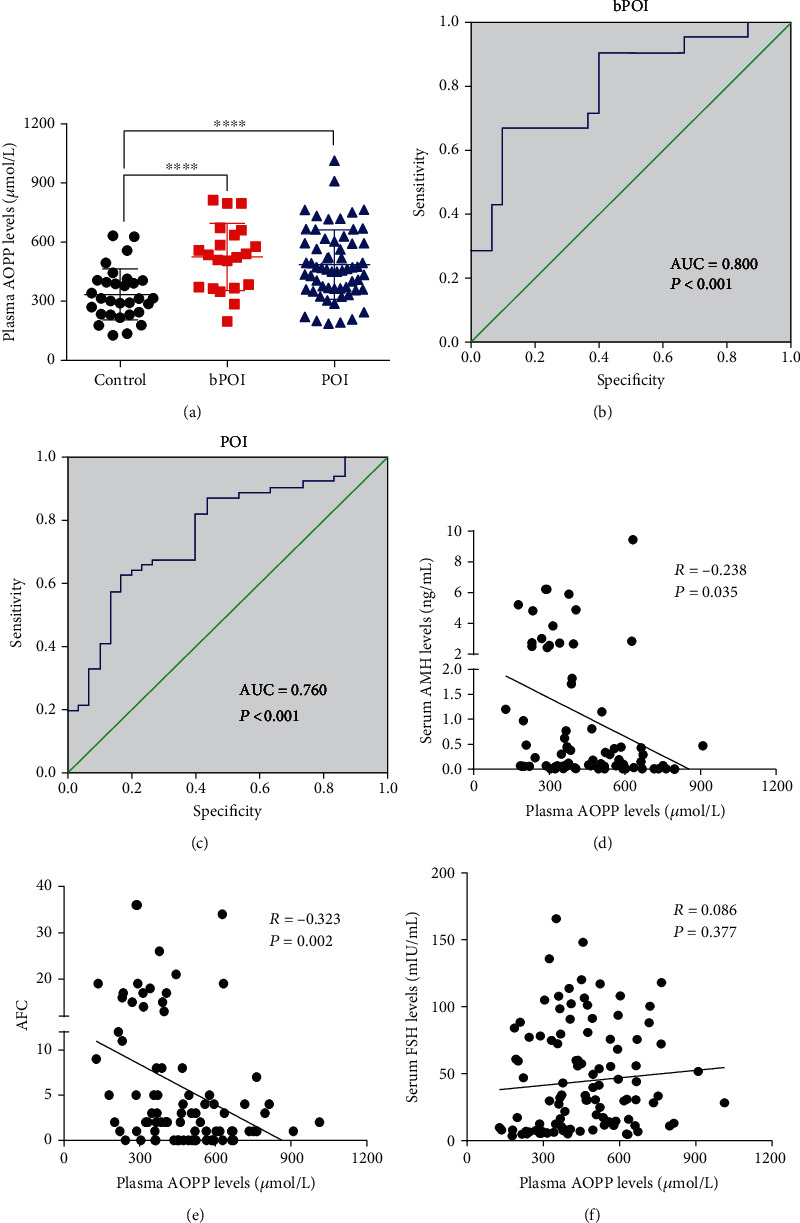
AOPP accumulation in the plasma of POI and bPOI patients. (a) Plasma AOPP levels were increased in both bPOI (*n* = 21) and POI (*n* = 61) patients compared to control subjects (*n* = 30). (b) ROC curve analysis of plasma AOPP levels to distinguish bPOI from normal-ovarian reserve controls. (c) ROC curve analysis of plasma AOPP levels to distinguish POI from normal-ovarian reserve controls. (d) Negative correlation between AOPP levels and AMH levels. (e) Negative correlation between AOPP levels and AFC. (f) There was no correlation between plasma AOPP levels and FSH levels. ∗∗∗∗*P* < 0.0001 vs. the control group.

Plasma from 61 POI patients, 21 bPOI patients, and 30 controls was collected to measure AOPP levels. The patients' clinical characteristics are summarized in Table 1. Plasma AOPP concentrations were significantly higher in both POI and bPOI patients than in age- and BMI-matched controls (Figure 1(a)). As shown in Figures 1(b) and 1(c), the area under the receiver operating characteristic (ROC) curve (AUC) for diagnosis was 0.760 (95% confidence interval (CI): 0.657–0.863, sensibility: 0.667, specificity: 0.900, *P* < 0.001) for POI patients and 0.800 (95% CI: 0.674–0.926, sensibility: 0.623, specificity: 0.833, *P* < 0.001) for bPOI patients. Pearson correlation analysis revealed that AOPP plasma levels were negatively correlated with AMH levels (Figure 1(d)) and AFC (Figure 1(e)) but were unrelated to FSH levels (Figure 1(f)).

### 3.2. AOPPs Induce G1/G0-Phase Arrest in KGN Cells

**Figure 2:**
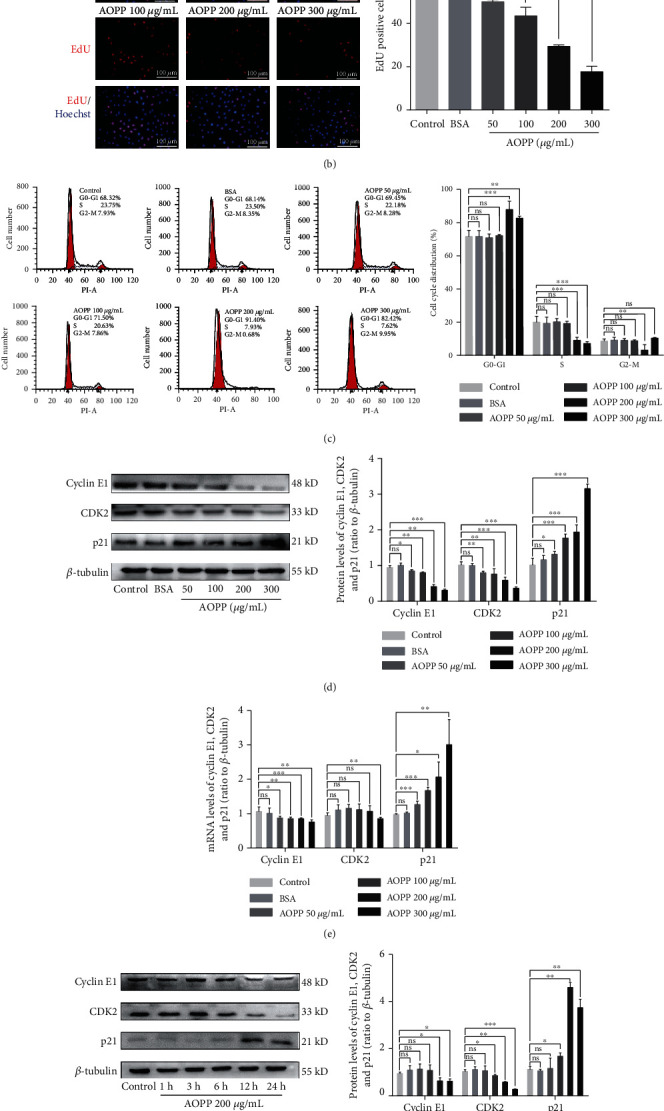
AOPP treatment induced G1/G0-phase cell cycle arrest in KGN cells. (a) The results of CCK-8 assays showed that AOPP treatment markedly decreased KGN cell proliferation in a concentration-dependent manner. (b) EdU assays indicated that AOPP treatment markedly decreased KGN cell proliferation. (c) Flow cytometry revealed an increased proportion of KGN cells in the G1/G0 phase and a decreased proportion of KGN cells in the S phase upon treatment with AOPPs at a concentration of 200 or 300 *μ*g/mL. (d) Reduced cyclin E1 and CDK2 protein expression and increased p21 protein expression occurred in KGN cells after AOPP treatment in a concentration-dependent manner. (e) Reduced cyclin E1 and CDK2 mRNA expression and increased p21 mRNA expression were found in KGN cells after AOPP treatment in a concentration-dependent manner. (f) Reduced cyclin E1 and CDK2 protein expression and increased p21 protein expression occurred in KGN cells after AOPP treatment in a time-dependent manner. (g) Reduced cyclin E1 and CDK2 mRNA expression and increased p21 mRNA expression were found in KGN cells after AOPP treatment in a time-dependent manner. ns: *P* > 0.05, ∗: *P* < 0.05, ∗∗: *P* < 0.01, ∗∗∗: *P* < 0.001 vs. the control group.

To assess the effects of AOPPs on cell proliferation in granulosa cells, KGN cells were treated with different AOPP concentrations ranging from 50 to 300 *μ*g/mL for 12 h to 48 h. The results of CCK-8 (Figure 2(a)) and EdU assays (Figure 2(b)) showed that AOPP treatment markedly decreased cell proliferation. hLGCs were also cultured to assess whether AOPP treatment affects the activity of normal human granulosa cells. CCK8 assays revealed that treatment with 50, 100, 200, or 300 *μ*g/mL AOPPs for 48 h led to a significant decline in hLGC activity (Supplementary Figure 1). To explore the potential mechanisms by which AOPPs affected granulosa cell proliferation, we studied the cell cycle distribution of KGN cells by flow cytometry. An increased proportion of KGN cells in the G1/G0 phase and a decreased proportion of KGN cells in the S phase were observed in the groups treated with 200 or 300 *μ*g/mL AOPPs (Figure 2(c) and Supplementary Table 1). Cyclin E1, cyclin-dependent kinase (CDK) 2, and p21 are key regulators of the G1-to-S cell cycle phase transition [22]. Exposure of KGN cells to AOPPs resulted in downregulation of cyclin E1 and CDK2 expression and upregulation of p21 expression at both the protein (Figures 2(d) and 2(f)) and mRNA (Figures 2(e) and 2(g)) levels in a concentration- and time-dependent manner. These results demonstrate that AOPPs markedly inhibit granulosa cell proliferation by inducing G0/G1-phase cell cycle arrest.

### 3.3. AOPPs Induce Intracellular ROS Generation via NADPH Oxidase Activation in KGN Cells

**Figure 3:**
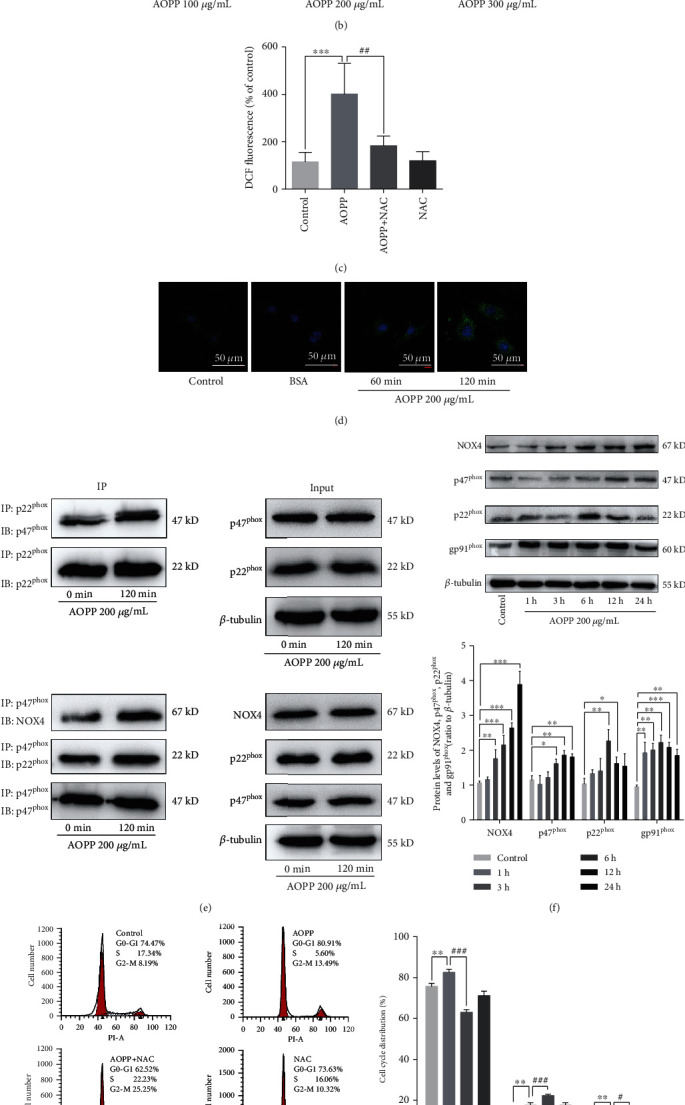
AOPPs induced intracellular ROS generation via NADPH oxidase activation. (a) A dichlorofluorescein (DCF) fluorescence assay showed that 200 *μ*g/mL AOPP administration significantly increased ROS production in KGN cells in a dose-dependent manner, whereas native BSA had no effect. (b) Fluorescence microscopy images using DCF revealed that AOPP induced ROS production in KGN cells in a dose-dependent manner. (c) Pretreatment with 2 mM NAC significantly attenuated the AOPP-triggered increases in intracellular ROS levels. (d) p47^phox^ presented membrane translocation 60 min or 120 min after 200 *μ*g/mL AOPP administration. (e) AOPPs enhanced the interactions of p47^phox^ with the membrane subunit p22^phox^ and of p22^phox^ with NOX4. (f) The protein levels of the NADPH oxidase subunits p47^phox^, p22^phox^, gp91^phox^, and NOX4 were increased after AOPP stimulation. (g) Flow cytometry showed that pretreatment with 2 mM NAC alleviated the AOPP-induced increase in the proportion of cells in the G1/G0 phase. (h) Cyclin E1 and CDK2 protein expression were higher, and p21 protein expression was lower after 2 mM NAC pretreatment and subsequent AOPP administration than after AOPP administration alone. ns: *P* > 0.05, ∗: *P* < 0.05, ∗∗: *P* < 0.01, ∗∗∗: *P* < 0.001 vs. the control group; ns: *P* > 0.05, #: *P* < 0.05, ##: *P* < 0.01 vs. the AOPP group.

A previous study demonstrated that AOPP can induce intracellular ROS production by activating NADPH oxidase [10]. We examined intracellular ROS levels in AOPP-treated KGN cells using a DCFH-DA fluorescence assay. As shown in Figures 3(a) and 3(b), AOPP administration significantly increased ROS production in KGN cells in a dose-dependent manner, whereas native BSA had no effect. Pretreatment with NAC, an ROS scavenger, significantly attenuated the AOPP-triggered induction of intracellular ROS production (Figure 3(c)).

NADPH oxidase activity was evaluated in KGN cells after AOPP stimulation to verify whether NADPH oxidase is the source of ROS generation. p47^phox^, a key cytoplasmic component of NADPH oxidase, presented membrane translocation 60 min or 120 min after AOPP administration (Figure 3(d)). AOPP enhanced the interactions of p47^phox^ with the membrane subunit p22^phox^ and of p22^phox^ with NOX4 (Figure 3(e)) and increased p47^phox^, p22^phox^, gp91^phox^, and NOX4 expression (Figure 3(f)).

Furthermore, to evaluate whether intracellular ROS accumulation is required for AOPP to suppress cell cycle progression in KGN cells, we pretreated cells with 2 mM NAC for 1 h before exposing the cells to AOPP. NAC pretreatment alleviated AOPP-induced G1/G0-phase arrest (Figure 3(g)), decreased cyclin E1 and CDK2 expression, and increased p21 expression (Figure 3(h)). These findings confirm that AOPP induced G1/G0-phase arrest in KGN cells is dependent on intracellular ROS generation activated by NADPH oxidase.

### 3.4. AOPP-Induced G1/G0-Phase Arrest in KGN Cells Is Mediated by the ROS-JNK/p38 MAPK-p21 Pathway

**Figure 4:**
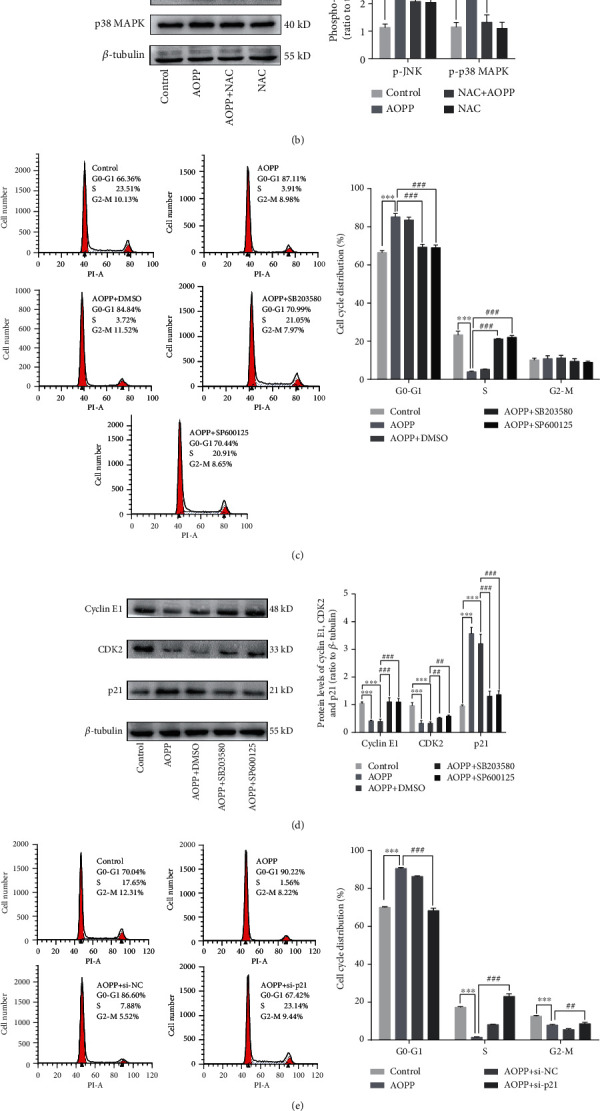
AOPP-induced G1/G0-phase arrest in KGN cells is mediated by the ROS-JNK/p38 MAPK-p21 pathway. (a) Western blotting revealed that p38 MAPK and JNK phosphorylation was significantly increased 60 min after 200 *μ*g/mL AOPP administration. (b) Pretreatment with 2 mM NAC significantly decreased the levels of phosphorylated p38 MAPK and JNK. (c) Flow cytometry showed that pretreatment with 10 nM SB203580 and 10 nM SP600125 for 1 h could attenuate the AOPP-induced increase in the proportion of G1/G0-phase cells. (d) Western blotting also showed that cyclin E1 and CDK2 protein expression was increased and that p21 protein expression was decreased after pretreatment with 10 nM SB203580 and 10 nM SP600125 compared to AOPP administration alone. (e) The G1/G0 cell cycle arrest caused by AOPPs could be prevented by pretreatment with 50 nM si-p21. (f) Pretreatment with 50 nM si-p21 significantly decreased p21 protein expression and increased cyclin E1 and CDK2 protein levels. ns: *P* > 0.05, ∗: *P* < 0.05, ∗∗: *P* < 0.01, ∗∗∗: *P* < 0.001 vs. the control group; ns: *P* > 0.05, #: *P* < 0.05, ##: *P* < 0.01, ###: *P* < 0.001 vs. the AOPP group.

The intracellular MAPK pathway is a well-known ROS downstream signaling pathway, including JNK, p38 MAPK, and extracellular signal-regulating kinase 1/2 (ERK1/2). To test whether the MAPK pathway is involved in AOPP-induced G1/G0-phase arrest in KGN cells, we measured the protein phosphorylation of MAPKs after AOPP treatment. As shown in Figure 4(a), AOPP treatment for 60 min, 120 min, and 180 min markedly increased JNK and p38 MAPK phosphorylation. NAC significantly blocked JNK and p38 MAPK phosphorylation (Figure 4(b)), indicating that the JNK and p38 MAPK activation was ROS-dependent. However, staining for phosphorylated ERK1/2 did not significantly increase from 30 to 180 min (Supplementary Figure 2).

To confirm the roles of activated JNK and p38 MAPK in AOPP-induced KGN G1/G0-phase arrest, KGN cells were pretreated with 10 nM SP600125 (a JNK inhibitor) and 10 nM SB203580 (a p38 MAPK inhibitor) 1 h prior to AOPP exposure. SB203580 blocked p38 MAPK phosphorylation but did not affect the expression of p38 MAPK. SP600125 remarkably inhibited JNK phosphorylation but did not have an effect on the JNK protein level (Supplementary Figure 3). As shown in Figures 4(c) and 4(d), compared to AOPP treatment alone or dimethyl sulphoxide (DMSO) pretreatment, SP600125 and SB203580 pretreatment decreased the proportion of cells in the G1/G0 phase, increased cyclin E1 and CDK2 expression, and decreased p21 expression.

Finally, we investigated the p21-dependent influences of AOPPs on cell cycle arrest. As expected, p21 siRNA transfection reduced p21 protein expression and increased cyclin E1 and CDK2 protein expression (Figure 4(f)). p21 siRNA transfection also significantly alleviated AOPP-induced G1/G0-phase arrest (Figure 4(e)). All the above results suggest that AOPP-induced G1/G0-phase arrest in KGN cells is mediated by the ROS-JNK/p38 MAPK-p21 pathway.

### 3.5. Chronic AOPP Loading In Vivo Increases Atretic Follicle Numbers in SD Rats

**Figure 5:**
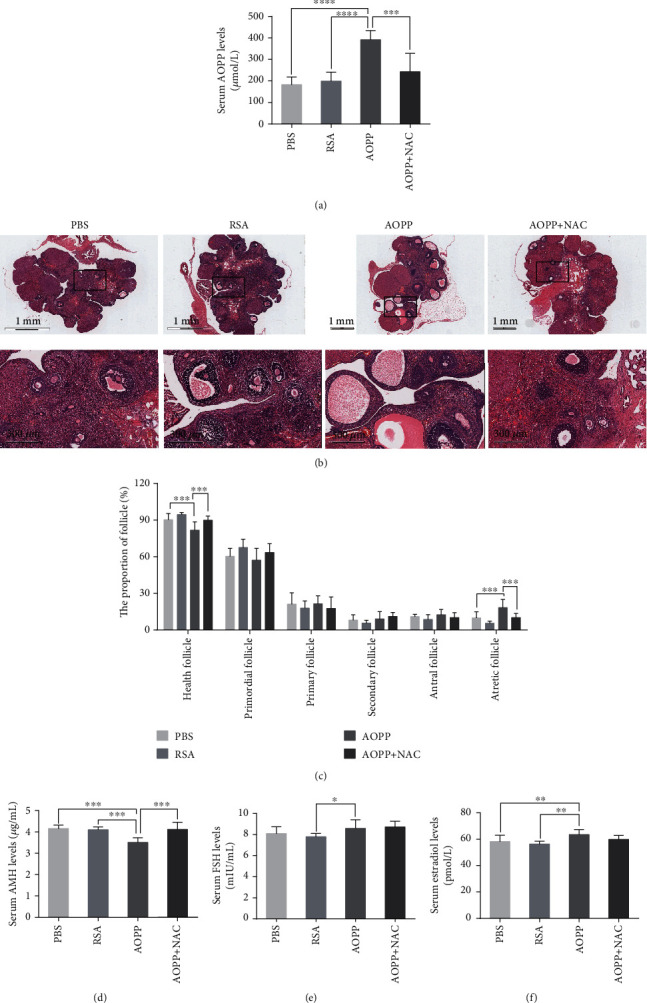
Chronic AOPP loading exacerbates follicular atresia in female rats. (a) Serum AOPP levels were significantly higher in the AOPP group than in the other groups (*n* = 9 per group). (b) Representative images of H&E staining of rat ovaries in different groups. Magnified images are shown in the bottom line. (c) Morphological analysis indicated that there were more atretic follicles in the AOPP group than in the PBS group or the RSA group, while NAC treatment alleviated AOPP-induced follicular atresia (*n* = 9 per group). (d) Significantly lower AMH levels were found in the AOPP group than in the PBS, RSA, and AOPP+NAC groups (*n* = 9 per group). (e) Serum FSH levels in the AOPP group were higher than those in the RSA group but were not different from those in the PBS and AOPP+NAC groups (*n* = 9 per group). (f) ELISA indicated that serum estradiol levels in the AOPP group were higher than those in the PBS group or the RSA group (*n* = 9 per group). ∗: *P* < 0.05, ∗∗: *P* < 0.01, ∗∗∗: *P* < 0.001, ∗∗∗∗: *P* < 0.0001.

To investigate the potential effect of chronic AOPP loading on ovarian function, we treated SD rats with AOPPs daily via intraperitoneal injection for 12 weeks. As expected, daily intraperitoneal AOPP injection markedly increased serum AOPP levels (Figure 5(a)). An increased proportion of atretic follicles was found in the AOPP group compared with the PBS group or the RSA group, but NAC treatment attenuated AOPP-induced follicular atresia (Figures 5(b) and 5(c)). Consistent with the histological results, hormone measurements showed that 12 weeks of AOPP exposure reduced serum AMH levels (Figure 5(d)). However, we observed only slight elevations in serum FSH levels in the AOPP group compared to the RSA group and similar elevations in serum estradiol levels in the AOPP group compared to the PBS group and the RSA group (Figures 5(e) and 5(f)).

**Figure 6:**
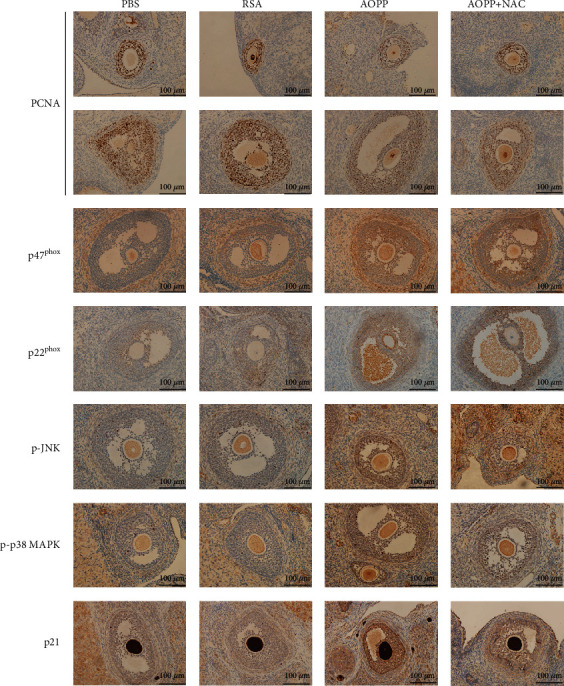
AOPP inhibited granulosa cell proliferation mediated by the ROS-JNK/p38 MAPK-p21 pathway in vivo. Representative images of immunohistochemical staining for PCNA, p47^phox^, p22^phox^, p-JNK, p-p38 MAPK, and p21 in granulosa cells of rats in different groups.

We next evaluated whether granulosa cell proliferation was suppressed by AOPPs in vivo. Immunohistochemical studies showed that chronic AOPP loading downregulated PCNA expression and upregulated p21 expression in granulosa cells (Figure 6). AOPP administration also activated JNK/p38 MAPK phosphorylation and NADPH oxidase in vivo (Figure 6). NAC administration significantly attenuated AOPP-induced suppression of cell proliferation and inhibited JNK/p38 MAPK phosphorylation (Figure 6).

## 4. Discussion

AOPPs are novel markers of oxidized proteins that circulate for hours to days in the blood and reflect the degree of oxidative imbalance in patients with multiple chronic inflammatory diseases [13], including atherosclerosis [23], chronic kidney disease [24], PCOS [25], and preeclampsia [26]. In this study, we first found that plasma AOPP levels were significantly elevated in both women with overt POI and women with bPOI compared to age-matched women with normal ovarian reserve. Moreover, a negative correlation between AMH/AFC and AOPP levels was observed in our study. Our results provide preliminary evidence that a high AOPP level has diagnostic value in POI and bPOI patients.

POI is usually regarded as a pathological condition of premature menopause. Cakir et al. found that plasma AOPP levels were higher in naturally postmenopausal women than in their premenopausal peers but found no significant differences in plasma AOPP levels between 40- to 49-year-old postmenopausal women and 50- to 59-year-old postmenopausal women [27]. However, another study failed to find any differences in AOPP levels between pre- and postmenopausal women [28]. In contrast to natural menopause, POI is aetiologically heterogeneous, the known causes of which include genetics, iatrogenesis, autoimmunity, and infection. Nevertheless, most cases of POI are idiopathic, and the principal mechanisms of POI remain obscure. Recently, some researchers have proposed that chronic low-grade inflammation may be a major contributor to the pathogenesis of POI [29]. Various inflammatory indicators and oxidative markers, including the neutrophil to lymphocyte ratio [29], platelet-activating factor [30], MIP-1*α*, CXCL8, IP-10, and eotaxin-1 [31], have been found to be increased in patients with POI. Previous studies also indicated that patients with POI had a high risk of atherosclerosis, osteoporosis, and Alzheimer's disease [1], which have also been demonstrated to be associated with chronic inflammation. Our results indicating that AOPPs accumulate in the plasma of bPOI and POI patients add to the growing evidence that chronic inflammation and oxidative stress may participate in the pathogenesis of POI. AMH and AFC have been recognized as the preferred biomarkers of ovarian reserve [32], and thus, the negative correlation between the plasma AOPP level and AMH or AFC further suggests that AOPP accumulation is closely related to the ovarian reserve function and has the potential to be a novel disease biomarker of POI.

Previous observations have revealed that AOPPs are not only oxidative markers but also pathogenic mediators in various disorders. AOPPs can exacerbate cardiomyocyte death, renal fibrosis, and preosteoblast apoptosis, thus, participating in ischaemic heart disease, chronic kidney disease, and osteoporosis [10, 33, 34]. Recently, Liu et al. demonstrated that the AOPP challenge could promote endometrial epithelial cell proliferation and migration and suppress apoptosis, thus, contributing to endometriosis [35]. High expression of placental AOPPs in preeclampsia can affect trophoblast cell endocrine function and inhibit trophoblast cell invasive capacity [36]. However, whether AOPPs play a role in the pathogenesis of POI is unclear. In our previous works, we tried to explore the aetiology of POI from genetics and epigenetics perspectives [20, 37], but we could not completely explain the occurrence and mechanisms of POI from only these two viewpoints. Therefore, in this study, we investigated the detrimental effects of AOPPs on granulosa cells and ovarian function to understand the mechanisms of AOPPs in POI.

Granulosa cells play a crucial role in ovarian function maintenance, and suppression of granulosa cell proliferation contributes to POI development [38]. Follicular development relies on orchestrated crosstalk between oocytes and granulosa cells [39]; thus, proliferation inhibition in granulosa cells may deprive follicular oocytes of nutrients, growth factors, and survival factors [40]. In addition, dysfunction of granulosa cells can result in follicular atresia, the major event responsible for oocyte elimination, leading to the onset of POI. Various ROS triggers have been revealed to induce cell cycle arrest, apoptosis, and estradiol biosynthesis impairment in granulosa cells [41–43], and some antioxidants, such as dehydroepiandrosterone (DHEA), melatonin, and resveratrol, can ameliorate excess ROS-induced damage by reducing ROS production to improve ovarian reserve [44–46]. Recently, the expression levels of antioxidant genes such as IDH1 and NDUFB10 were found to be associated with granulosa cell ageing through single-cell RNA sequencing [47]. MicroRNA-15b was also found to inhibit *α*-Klotho expression, reducing granulosa cell ROS-scavenging ability, and ultimately causing POI [8]. This evidence demonstrates that oxidant/antioxidant homeostasis plays a crucial role in granulosa cell function and follicle loss. This study is the first to conclude that accumulated AOPP triggered intracellular ROS generation via NADPH oxidase and then inhibited granulosa cell proliferation. In vivo, dysfunctional granulosa cells could result in follicular atresia, contributing to the progression of POI, while NAC can reverse AOPP-induced function in granulosa cells.

NAC, known as a potent antioxidant, is a thiol-containing precursor of glutathione that scavenges ROS and has been widely used in clinics for over 50 years [48]. A strong ovary-protecting effect of NAC has been demonstrated in the contexts of ionizing radiation-induced ovarian failure [48], ovarian torsion [49], ovarian grafts [50], and in Bmi1-knockout female mice [38]. A systematic review has reported that compared to a placebo, NAC significantly improves the rates of live births and spontaneous ovulation in women with PCOS [51]. A previous study has also shown that pretreatment with NAC reverses AOPP-induced S-phase arrest in hepatocytes and prevents liver regeneration impairment after partial hepatectomy in model rats [19]. Our observations suggest that NAC can reduce ROS overproduction induced by the AOPP challenge, thus, exerting a protective effect against AOPP-induced granulosa cell arrest, and might therefore be a novel effective clinical drug for delaying follicle depletion in POI.

The MAPK pathway has been widely studied as a downstream signaling pathway of ROS that crucially participates in cellular proliferation, differentiation, and survival. Our previous studies revealed that MAPK pathway activation after MALAT1 knockdown leads to G0/G1-phase cell cycle arrest mediated by increased p21 expression in granulosa cells [52]. In the present study, we found increased p38 MAPK and JNK phosphorylation levels after treatment of KGN cells with AOPPs, and the JNK inhibitor SP600125 or p38 MAPK inhibitor SB203580 was effective in blunting AOPP-induced cell cycle arrest in granulosa cells, which is consistent with the findings of previous studies on intestinal epithelial cells [18], cardiomyocytes [53], keratinocytes [54], and preosteoblasts [10]. However, Sun et al. found that AOPP-induced S-phase arrest was independent of the MAPK pathway in hepatocytes [19]. Moreover, the observation of JNK and p38 MAPK phosphorylation, but not ERK1/2 phosphorylation, in our study suggests that AOPP-triggered MAPK pathway activation is dependent upon the particular organ and cell contexts.

p21 was the first discovered CDK inhibitor, also known as CDKN1A. p21 serves as a negative cell cycle regulator by inhibiting CDK activity via interaction with its N-terminal domain or via interference with CDK phosphorylation. Moreover, it is believed that the regulation of G1/G0 arrest by p21 is mediated by inhibition of cyclin E/CDK2 and cyclin A/CDK2 activity [55]. Our results showed that p21 was aberrantly activated after AOPP treatment and that si-p21 suppressed G1/G0 arrest and attenuated the increases in p21 protein levels, suggesting that AOPPs are involved in G1/G0 arrest via p21 activation. In addition, we found that upstream p38 MAPK/JNK phosphorylation was involved in AOPP-induced p21 activation in KGN cells. Recent investigations have revealed that MAPKs can enhance p21 mRNA transcription [56], which is consistent with our findings. Furthermore, p27 is another well-known CDK inhibitor that has also been reported to participate in AOPP-triggered G0/G1 arrest in intestinal epithelial cells [18]; however, in our study, p27 was not a key regulator of G1 progression after AOPP treatment in KGN cells.

## 5. Conclusions

In conclusion, we found that plasma AOPP levels are increased in POI patients and are associated with ovarian reserve indicators, including AMH levels and AFC. AOPPs can suppress cell cycle progression in granulosa cells both in vitro and in vivo. In vivo, this inhibition of granulosa cell proliferation may lead to follicular atresia. In addition, we have provided mechanistic evidence that AOPP-induced cell cycle arrest in granulosa cells is regulated through the ROS-JNK/p38 MAPK-p21 pathway. Thus, AOPPs may be a valuable novel biomarker of POI, and targeting AOPPs may be an effective therapeutic strategy for the management of POI.

## Figures and Tables

**Table 1:** Clinical characteristics of patients with premature ovarian insufficiency, patients with biochemical premature ovarian insufficiency and controls.

Variable	Control	bPOI	POI	*P* value
*n*	30	21	61	—
Age (year)	31.15 ± 4.54	31.95 ± 4.85	30.93 ± 4.88	0.681
AMH (ng/mL)	3.83 ± 2.07	0.36 ± 0.33	0.18 ± 0.03	<0.001
AFC	17.87 ± 8.248	2.94 ± 2.13	1.58 ± 1.91	<0.001
Basal FSH (mIU/mL)	6.55 ± 1.41	15.29 ± 5.47	70.87 ± 33.41	<0.001
Basal LH (mIU/mL)	5.17 ± 1.88	8.45 ± 1.84	35.82 ± 20.54	<0.001
Basal estradiol (pg/mL)	48.31 ± 21.86	58.95 ± 66.87	41.08 ± 84.74	0.551
Basal progesterone (ng/mL)	0.45 ± 0.41	0.27 ± 0.24	0.37 ± 0.38	0.283

The data are presented as the mean ± standard deviation, and *P* < 0.05 was considered to indicate statistical significance. bPOI: biochemical premature ovarian insufficiency; POI: premature ovarian insufficiency; AMH: anti-Müllerian hormone; AFC: antral follicle count; FSH: follicle-stimulating hormone; LH: luteinizing hormone.

## Data Availability

The data used to support the findings of this study are available from the corresponding author upon request.

## Supplementary Materials

Supplementary Materials Supplementary Table 1: comparison of the proportion of cells in the G1/G0 phase in different groups treated with a series of AOPP concentrations. Supplementary Figure 1: AOPP inhibited cell activity in human luteinized granulosa cells. Supplementary Figure 2: the effect of AOPPs on ERK 1/2 and p-ERK 1/2 protein expression. Supplementary Figure 3: the effect of SB203580 and SP600125 on p-p38 MAPK, p38 MAPK, p-JNK, and JNK protein expression.
