# Supplementary material for: Advanced Oxidation Protein Products Induce G1/G0-Phase Arrest in Ovarian Granulosa Cells via the ROS-JNK/p38 MAPK-p21 Pathway in Premature Ovarian Insufficiency
Source: Oxid Med Cell Longev. 2021 Jul 27;2021:6634718. doi: 10.1155/2021/6634718 (PMC8337115; doi:10.1155/2021/6634718)
Supplement: Supplementary Materials — Supplementary Table 1: comparison of the proportion of cells in the G1/G0 phase in different groups treated with a series of AOPP concentrations. Supplementary Figure 1: AOPP inhibited cell activity in human luteinized granulosa cells. Supplementary Figure 2: the effect of AOPPs on ERK 1/2 and p-ERK 1/2 protein expression. Supplementary Figure 3: the effect of SB203580 and SP600125 on p-p38 MAPK, p38 MAPK, p-JNK, and JNK protein expression. [file 6634718.f1.docx]

**Supplementary table 1. Comparison of the proportion of cells in G1/G0 phase in the different groups treated with a series of AOPP concentrations**

| **Group 1** | **Group 2** | **P value** |
| --- | --- | --- |
| control | BSA | > 0.05 |
| control | AOPP 50 μg/mL | > 0.05 |
| control | AOPP 100 μg/mL | > 0.05 |
| control | AOPP 200 μg/mL | < 0.001 |
| control | AOPP 300 μg/mL | < 0.01 |
| BSA | AOPP 50 μg/mL | > 0.05 |
| BSA | AOPP 100 μg/mL | > 0.05 |
| BSA | AOPP 200 μg/mL | < 0.001 |
| BSA | AOPP 300 μg/mL | < 0.01 |
| AOPP 50 μg/mL | AOPP 100 μg/mL | > 0.05 |
| AOPP 50 μg/mL | AOPP 200 μg/mL | < 0.001 |
| AOPP 50 μg/mL | AOPP 300 μg/mL | < 0.01 |
| AOPP 100 μg/mL | AOPP 200 μg/mL | < 0.001 |
| AOPP 100 μg/mL | AOPP 300 μg/mL | < 0.01 |
| AOPP 200 μg/mL | AOPP 300 μg/mL | > 0.05 |

BSA, bovine serum albumin; AOPPs, advanced oxidation protein products.


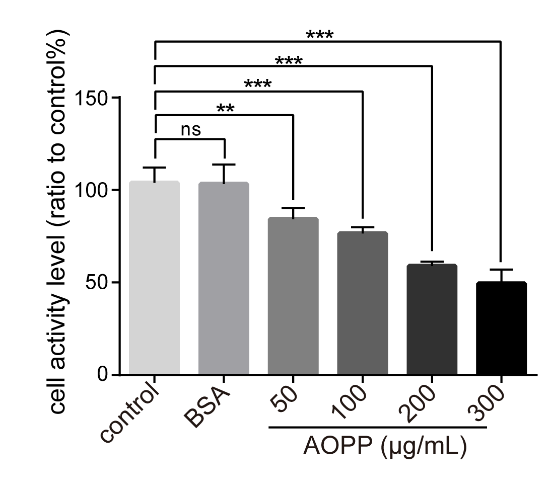


**Supplementary figure 1. AOPP inhibited cell activity in human luteinized granulosa cells.**

The results of CCK-8 assays showed that AOPP treatment for 48 h markedly decreased human luteinized granulosa cell activity in a concentration-dependent manner. ns: P > 0.05, **: P < 0.01, ***: P < 0.001 vs. the control group.


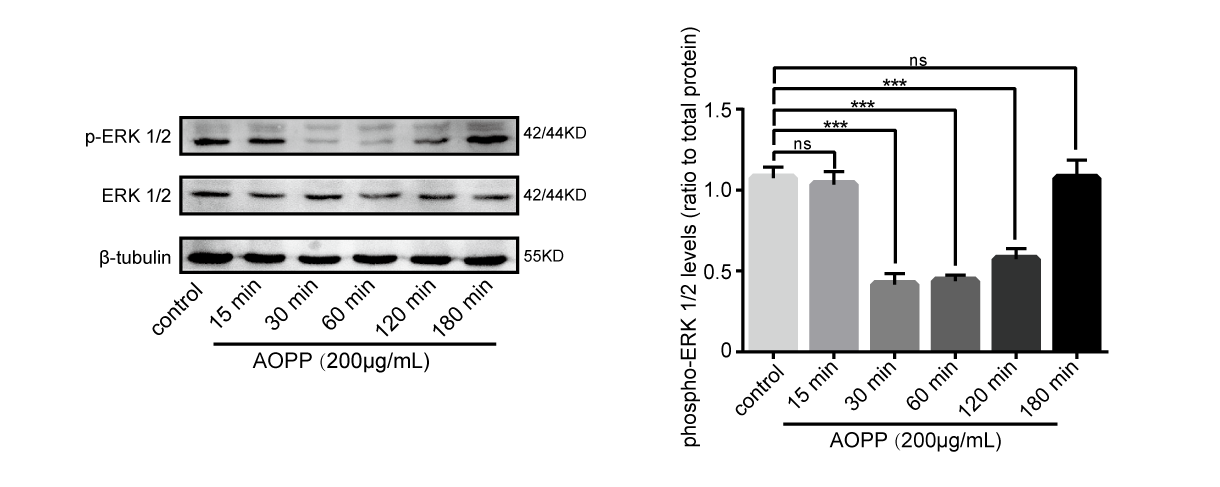


**Supplementary figure 2. The effect of AOPPs on ERK 1/2 and p-ERK 1/2 protein expression.**

Western blotting revealed that the phospho-ERK 1/2 levels were decreased in KGN cells treated with AOPP for 30 min, 60 min and 120 min, and the phospho-ERK 1/2 levels returned to the control levels after AOPP treatment of KGN cells for 180 min. ns: P > 0.05, ***: P < 0.001 vs. the control group.


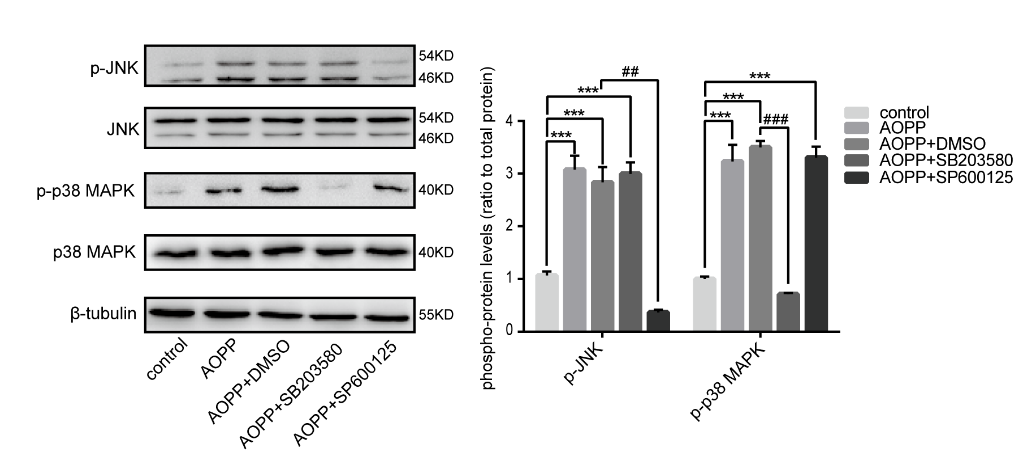


**Supplementary figure 3. The effect of SB203580 and SP600125 on p-p38 MAPK, p38 MAPK, p-JNK and JNK protein expression.**

Western blotting revealed that SB203580 blocked p38 MAPK phosphorylation but did not affect the p38 MAPK expression level. SP600125 remarkably inhibited JNK phosphorylation but did not have an effect on the JNK protein level. ***: P < 0.001 vs. the control group; ##: P < 0.01, ###: P < 0.001 vs. the AOPP+DMSO group.
